# Reduced β-amyloid pathology in an APP transgenic mouse model of Alzheimer’s disease lacking functional B and T cells

**DOI:** 10.1186/s40478-015-0251-x

**Published:** 2015-11-11

**Authors:** Claudia Späni, Tobias Suter, Rebecca Derungs, Maria Teresa Ferretti, Tobias Welt, Fabian Wirth, Christoph Gericke, Roger M. Nitsch, Luka Kulic

**Affiliations:** Division of Psychiatry Research, University of Zurich, Wagistrasse 12, 8952 Schlieren, Switzerland; Department of Neurology, Section of Neuroimmunology and MS Research, University Hospital Zurich, 8091 Zurich, Switzerland; Zurich Center for Integrative Human Physiology (ZIHP), University of Zurich, Rämistrasse 71, 8006 Zurich, Switzerland; Neuroscience Center Zurich (ZNZ), Wintherthurerstrasse 190, 8057 Zurich, Switzerland

**Keywords:** Alzheimer’s disease, Adaptive immunity, Amyloid β-peptide, Rag2 knockout mice, Microglia

## Abstract

**Introduction:**

In Alzheimer’s disease, accumulation and pathological aggregation of amyloid β-peptide is accompanied by the induction of complex immune responses, which have been attributed both beneficial and detrimental properties. Such responses implicate various cell types of the innate and adaptive arm of the immunesystem, both inside the central nervous system, and in the periphery. To investigate the role of the adaptive immune system in brain β-amyloidosis, PSAPP transgenic mice, an established mouse model of Alzheimer’s disease, were crossbred with the recombination activating gene-2 knockout (Rag2 ko) mice lacking functional B and T cells. In a second experimental paradigm, aged PSAPP mice were reconstituted with bone marrow cells from either Rag2 ko or wildtype control mice.

**Results:**

Analyses from both experimental approaches revealed reduced β-amyloid pathology and decreased brain amyloid β-peptide levels in PSAPP mice lacking functional adaptive immune cells. The decrease in brain β-amyloid pathology was associated with enhanced microgliosis and increased phagocytosis of amyloid β-peptide aggregates.

**Conclusion:**

The results of this study demonstrate an impact of the adaptive immunity on cerebral β-amyloid pathology in vivo and suggest an influence on microglia-mediated amyloid β-peptide clearance as a possible underlying mechanism.

**Electronic supplementary material:**

The online version of this article (doi:10.1186/s40478-015-0251-x) contains supplementary material, which is available to authorized users.

## Introduction

Despite the long-held view of the central nervous system (CNS) as an immune-privileged site, the importance of local and systemic immunological responses in neurodegenerative brain diseases like Alzheimer’s disease has been widely recognized in the past few years [1]. In Alzheimer’s disease, accumulation of misfolded amyloid β-peptide (Aβ) peptides plays a central role in disease pathogenesis and has been associated with a wide range of adverse reactions ultimately leading to neuronal dysfunction and neurodegeneration [2, 3]. Local immune responses to cerebral Aβ aggregates are a pathological hallmark of Alzheimer’s disease involving activation of cellular components of the brain’s innate immunity, such as microglia and astrocytes, and release of various immune molecules and inflammatory mediators, including cytokines, acute phase proteins and factors of the complement cascade, among others [4–7]. This activation of the brain’s innate immune system appears to be a double-edged sword as it has been related to both beneficial and detrimental consequences [8]. Microglia, the resident phagocytes of the CNS, for example, are able to bind to Aβ via various cell-surface receptors and promote phagocytosis and clearance of Aβ aggregates [5]. Their chronic activation in Alzheimer’s disease, however, has also been associated with disturbed clearance functions and an increased production of pro-inflammatory and neurotoxic factors, which may further promote neuronal injury and exacerbate neuronal loss [9].

In contrast to the activation of the brain’s innate immune system, the role of the adaptive immunity in cerebral β-amyloidosis appears to be much less prominent and is still incompletely understood. Consistent with a role of the humoral adaptive immunity in Alzheimer’s disease pathogenesis, misfolded Aβ peptides have been shown to induce specific B cell-mediated immune responses, with naturally occurring autoantibodies against Aβ being found in the cerebrospinal fluid (CSF) and blood of affected patients, as well as, healthy individuals [10–17]. Interestingly, these autoantibodies seem to share conformation-specific binding epitopes and the ability to promote microglia-mediated clearance of β-amyloid plaques, as well as to lower neurotoxicity [12, 18–20]. In support of these findings, Britschgi and colleagues demonstrated that naturally occurring autoantibodies against a broad range of amyloidogenic peptides can display neuroprotective activities [21], with similar findings being reported by other investigators [22, 23]. Apart from the involvement of B cell-mediated humoral immune responses, several authors have begun addressing the role of the cellular adaptive immunity in Alzheimer’s disease. Previous neuropathological analyses repeatedly confirmed the presence of T cells in post-mortem Alzheimer’s disease brains [14, 24–29]. Moreover, Aβ-reactive T cells were readily detectable in the peripheral blood of Alzheimer’s disease patients and healthy elderly subjects, in whom their proportion was significantly increased in comparison to middle-aged control individuals [30]. Analysis of the profile of specific lymphocyte subsets led to ambiguous findings. Richart-Salzburger, for example, reported a significant decrease of CD3^+^ CD8^+^ and CD19^+^ lymphocytes in the peripheral blood of Alzheimer’s disease patients, suggesting a general decline of the adaptive immunity in disease pathogenesis [31]. Other authors, in contrast, observed significant increases in activated CD4^+^ and CD8^+^ T cell subsets in peripheral blood [32–35]. Recently, Lueg et al. also assessed alterations of T cell subsets in the intrathecal compartment of cognitively impaired and cognitively intact elderly subjects as well as healthy younger control individuals and found a significantly increased proportion of CD8^+^ lymphocytes in the CSF of patients with Alzheimer’s disease – but not in patients suffering from other dementias – which correlated with cognitive deficits [33]. Altogether, these results provided further evidence for an involvement of the adaptive immunity in Alzheimer’s disease.

In this study, we addressed the role of a congenital deficiency of the adaptive immune system on β-amyloid-related pathology *in vivo* and generated B and T cell-ablated APP transgenic mice by crossbreeding the PSAPP mice, en established mouse model of Alzheimer’s disease [36], with the recombination activating gene-2 knockout (Rag2 ko) mice lacking functional B and T lymphocytes [37]. In a second experiment, the effects of an acquired deficiency of the adaptive immunity were studied in lethally irradiated aged PSAPP mice reconstituted with Rag2 ko bone marrow. Our results from both experimental paradigms demonstrate an impact of functional adaptive immune cells on cerebral β-amyloid deposition *in vivo* and suggest an increased Aβ clearance, likely mediated by microglial cells, as a possible mechanism underlying reduced Aβ pathology in the absence of functional B and T cells.

## Materials and methods

### Animals and tissue processing

The Rag2 ko mouse lines [37] and the wildtype (WT) mouse line expressing the Ptprc^a^ (CD45.1 or Ly5.1) allele (Model 4007) were purchased from Taconic (Ry, Denmark). The PSAPP transgenic mice express a chimeric mouse/human amyloid precursor protein (Mo/HuAPP695swe) and a mutant human presenilin 1 (PS1-dE9) and were purchased from the Jackson Laboratory (Maine, USA) [36, 38]. All mice were on the C57Bl/6 (H2^b^) genetic background. Rag2 ko mice (Model RAGN12; immunophenotype H2^b^ (C57B/6), CD45.2) were crossed with the PSAPP mice (immunophenotype H2^b^ (C57B/6), CD45.2) to generate the four genotypes PSAPP/Rag2 ko, PSAPP, Rag2 ko and WT in the F2 generation. For adoptive bone marrow (BM) transfer experiments, Rag2 ko donor mice expressing the immunophenotype H2^b^ (C57B/6), CD45.1 were used (Model 461). All mice were housed in groups (two to five animals per cage in 396 × 215 × 172 mm cages (Indulab, Gams, Switzerland)) under specific pathogen free (SPF) conditions at 22 °C ±1 °C and a 12 h light–dark cycle beginning at 7:00 AM. The health status of animals was monitored twice a week except for the BM-transferred mice, which were monitored every second day during the first two weeks after irradiation. No major adverse events were observed. Water and food were provided *ad libitum* except for the time of cognitive testing. Environmental enrichment included bedding, one cardboard mouse house and a paper tissue as nesting material.

For all biochemical and histological analyses gender-balanced groups of mice were used. Mice were deeply anesthetized with ketamine/xylazine. Blood samples were collected from the right ventricle in ethylenediaminetetraacetic acid (EDTA)-coated tubes (Sarstedt, Sevelen, Switzerland) and centrifuged for plasma preparation at 2000 g for 10 min at 4 °C. Following the collection of blood, mice were transcardially perfused with ice-cold phosphate buffered saline (PBS) and their brains rapidly removed. Brains were dissected into two hemispheres with the left hemisphere being placed in 4 % (w/v) paraformaldehyde (PFA) in PBS, post-fixed overnight in the same medium at 4 °C and transferred into a 30 % sucrose solution (in PBS) for 72 h (cryoprotection). The right hemisphere was snap frozen and stored at −80 °C for later biochemical analysis.

All animal experiments were performed in compliance with Swiss law for care and use of animals (“455.163 Tierversuchsverordnung” 2010) and were approved by the veterinarian authorities of the Canton of Zurich (license numbers 079/2010 and 102/2013). All sections of this report adhere to the ARRIVE guidelines for reporting animal research [39]. A complete ARRIVE guidelines checklist is included.

### Protein extracts and Western blotting

Homogenization of brain tissue was performed using a glass Teflon homogenizer in fivefold wet weight amount of buffer A (100 mM Tris–HCl, 150 mM NaCl, Phosphatase Inhibitor Cocktails 2 + 3 (Sigma-Aldrich, Buchs, Switzerland) and Complete Protease Inhibitor Cocktail (Roche Diagnostics, Rotkreuz, Switzerland)). Supernatants were collected (=Tris fraction) after centrifugation at 100’000 g for 1 h. The pellets were rehomogenized in buffer A containing 2 % sodium dodecyl sulfate (SDS). Centrifugation was repeated and supernatants again were again collected (=SDS fraction). The resulting pellets were dissolved in 70 % formic acid (FA), sonicated two times for 30 s at 30 % power, ultracentrifuged at 100’000 g for 30 min, supernatants extracted, lyophilized and reconstituted in buffer A for further analysis. Extracts were separated by SDS-polyacrylamide gel electrophoresis, blotted onto nitrocellulose, blocked in Tris-buffered saline containing 5 % milk for 1 h at room temperature. Blots were incubated with the primary antibody overnight at 4 °C, then visualized via peroxidase-conjugated antibody and ECL reaction (Amersham Biosciences, Otelfingen, Switzerland). The primary rabbit polyclonal antibody anti-C-terminal APP (Sigma-Aldrich Chemie GmbH, Buchs, Switzerland) was used at a 1:2000 dilution. A mouse monoclonal anti-GAPDH antibody (Meridian Life Science, Memphis, USA) was used as internal loading control and for normalization of densitometric analyses of the immunoreactive bands (dilution 1:10’000). Image J software (rsb.info.nih.gov/ij/) was used for quantifications of the immunoreactive bands by densitometry of the scanned films under conditions of non-saturated signal.

### Meso Scale Discovery (MSD) analysis

Aβ40 and Aβ42 levels were measured in the above-mentioned brain homogenate fractions and plasma using an MSD 3plex multi-SPOT Aβ human kit (Gaithersburg, MD, USA) as previously described [40]. Plates were measured on an MSD SECTOR Imager 600 plate reader (Gaithersburg, MD, USA) after adding the MSD Read Buffer T (Gaithersburg, MD, USA). The MSD DISCOVERY WORKBENCH software (Version 3.0.17) (Gaithersburg, MD, USA) with Data Analysis Toolbox was used to calculate sample concentrations by comparing them against a standard curve.

### Histological analysis

PFA-fixed and cryoprotected hemibrains were cut continuously into 35 μm thick coronal slices at −20 °C using a microtome (Leica Jung HN40, Leica Microsystems AG, Heerbrugg, Switzerland) and stored at −20 °C in an anti-freeze solution (phosphate buffer 0.50 M in MilliQ water: ethyleneglycol: glycerol = 1.3:1:1) until use. For each staining six equidistant sections per mouse were analyzed from positions −1.94 mm to +1.70 mm from the bregma [41]. For immunohistochemistry, sections were first washed with PBS + 0.2 % Triton X-100, blocked with appropriate normal sera for 1 h and incubated with the primary antibody overnight at 4 °C. The following primary antibodies were used: Glial fibrillary acidic protein (GFAP; 1:400 dilution; rabbit polyclonal; Sigma-Aldrich Chemie GmbH, Buchs, Switzerland), Ionized calcium binding adaptor molecule 1 (Iba1; 1:500 dilution; rabbit polyclonal; Wako Chemicals, Osaka, Japan) and anti-β amyloid 1–16 (clone 6E10; 1:500 dilution; mouse monoclonal; BioLegend, Fell, Germany). Secondary antibodies were obtained from Jackson Immunoresearch Laboratories (West Grove, PA, USA). Thioflavin S staining was performed according to the standard protocols as previously described [42]. 4′,6-Diamidin-2-phenylindol (DAPI; Sigma-Aldrich Chemie GmbH, Buchs, Switzerland) was used as counterstaining agent. For quantitative image analysis, the cortex (including the cingulate, motor and somatosensory cerebral cortex) was analyzed on six equidistant coronal hemibrain sections from positions −1.94 mm to +1.70 mm from the bregma [41]. Mean values per mouse were calculated and included into statistical analysis. Average plaque numbers refer to single hemibrain sections. Quantitative image analysis was performed blinded using ImageJ software (rsb.info.nih.gov/ij/). For specific microglial analyses around amyloid plaques serial confocal Z-stack images, comprising an average of 30 to 40 cortical brain sections at 0.26 μm intervals, were acquired using a confocal laser scanning microscope (Leica TCS SP8, Leica Microsystems AG, Heerbrugg, Switzerland). On average, 12 cortical Thioflavin S-positive amyloid plaques per mouse were analyzed. Plaques of an average size between 20 and 30 μm were randomly selected by the blinded investigator between cortical layers II and V on six consecutive equidistant sections (2 plaques per section) from positions −1.94 mm to +1.70 mm from the bregma [41]. Counting of microglia was done in a diameter of 100 μm around the center of the amyloid plaque as previously described [43].

### Adoptive bone marrow transfer

For the generation of bone marrow (BM) chimeras, 12 month-old PSAPP transgenic recipient mice (CD45.2; 12 females and 7 males) were lethally irradiated with two doses of 600 rads each (second irradiation after 12 h). 3 month-old Rag2 ko (Model 461, Taconic, Ry, Denmark) and age-matched WT control donor mice (Model 4001, Taconic, Ry, Denmark) expressing the CD45.1 allele were sacrificed by CO_2_ inhalation, and bones (including femur, tibia and pelvis) were flushed with sterile PBS to obtain BM stem cells. A total of 5 x 10^6^ cells in 0.2 ml sterile Hank’s Buffered Salt Solution (HBSS, Life Technologies, Zug, Switzerland) were injected i.v. per animal in a randomized and gender-balanced fashion (10 PSAPP mice (including 6 females and 4 males) received Rag2 ko BM; 9 PSAPP mice (including 6 females and 3 males) received WT BM). BM reconstitution of recipient mice was carried out with BM from gender-matched donors. Autoclaved drinking water supplemented with antibiotics was provided for two weeks (2 g/l Neomycin sulphate; Sigma Aldrich). Reconstitution efficiency with CD45.1 positive cells was assessed on the day of sacrifice (6 months later) by flow cytometry of EDTA whole blood samples.

### Flow cytometry

Ammonium-Chloride-Potassium (ACK) lysis of red blood cells was performed on EDTA blood samples followed by HBSS and FACS buffer (HBSS, 2 % fetal calf serum (FCS), 10 mM EDTA, 0.01 % NaN_3_) washing steps. After using a live/dead exclusion dye (Molecular Probes, Eugene, OR, USA) an Fc blocking step (anti-mouse CD16/32, TruStain fcX™, Biolegend, CA, USA) was used to reduce non-specific binding. The cells were then stained for 15 min at 4 °C with surface marker-specific antibodies: anti-CD45 (PE conjugated, rat anti-mouse, clone 30-F11, BioLegend, Fell, Germany), anti-CD45.1 (PE conjugated, mouse anti-mouse, clone A20, BioLegend, Fell, Germany), anti-CD45-2 (FITC conjugated, mouse anti-mouse, clone 104, BioLegend, Fell, Germany), anti-CD11b (APC or APC-Cy7 conjugated, rat anti-mouse/human, clone M1/70, BioLegend, Fell, Germany), anti-CD4 (FITC or APC conjugated, rat anti-mouse, clone RM4-5, BioLegend, Fell, Germany), anti-Ly6C (FITC conjugated, rat anti-mouse, clone AL-21, BD Biosciences, Allschwil, Switzerland), anti-Ly6G (APC-conjugated, rat anti-mouse, clone 1A8, BioLegend, Fell, Germany) and anti-B220/CD45R (PerCP-Cy5.5 or APC-Cy7 conjugated, rat anti-mouse/human, clone RA3-6B2, BioLegend, Fell, Germany). After additional washing with FACS buffer samples were analyzed blinded using a BD LSRFortessa™ cell analyser (BD Biosciences, Allschwil, Switzerland). Single stainings and isotype control antibodies were included as controls.

### Cognitive-behavioral testing

A battery of validated tests was used to assess motoric and cognitive behavior of the mice. For all cognitive tests, the state-of-the-art ANY-maze Video Tracking System software (Stoelting Co., USA) was used as an automated system to track animal movements and completion of test specific tasks. All animals were tested naïve in a random order. The tested groups were balanced for gender. The experimenter was blinded during the time of testing.

#### Open Field

The Open Field test measures locomotor activity and anxiety. Mice were placed in the centre of a white box (49 x 49 x 33 cm) with indirect illumination (22 Lux) and their movements were tracked for 15 min. Distance travelled, average speed and time spent in the central (inner square 33 x 33 cm) versus peripheral zones of the open field were calculated using the ANY-maze software [44].

#### Novel Object Recognition Test (NORT)

This test measures memory retention and recognition memory in mice and was performed in accordance to published protocols [45]. The NORT has been designed as a two-trial task including a sample trial (ST) and a test trial (TT) and was performed following the Open Field test with a delay of 5 min in the same box. During ST, two similar objects were placed in the box, 34 cm apart (symmetrically). Each animal was allowed to explore the box for 5 min. The animals were considered to be exploring the object when the head of the animal was within a defined area of 12 cm in diameter around the object. The objects sizes ranged within diameters from 3–4 cm, so the animal was considered exploring the object when the animals head was near the object as close as 4 cm. After ST, mice were immediately returned to their holding cages and following a 10 min delay TT was carried out. One of the objects used during ST was replaced by a novel one differing in form and color before each mouse was placed back into the same box as during ST. The mice were then allowed to freely explore for 5 min and the time spent exploring each object was recorded. The ratio was calculated by dividing measures of the new object versus the old one.

#### Y-Maze

The Y-Maze was used to evaluate spatial working memory in mice and was carried out according to published protocols [40]. The Y-Maze consists of three horizontal arms (40 cm long, 11 cm wide and 21 cm high) symmetrically disposed at 120° to each other. Light was adjusted to 28 Lux. The mice were placed at one end of the first arm and then allowed to freely explore for 5 min. The mice were tracked recording arm entries and exploration time in each arm. The total number of arm entries was measured as an index of locomotor activity to rule out the interference of changes in motility with the parameters of learning and memory [46, 47]. The percentage alternation was calculated as the ratio of actual to possible alternations (defined as the total number of arm entries −2) x 100 %.

### Statistical analysis

The primary experimental outcome of our study included Aβ pathology (amyloid plaque pathology, Aβ levels) and glial pathology. Secondary outcome measures included cognitive performance and APP processing. Data analysis was performed using the SPSS software version 19.0 (16 M, Zurich, Switzerland). Extreme values according to SPSS explorative data analysis were excluded from further analysis. Tests for normal distribution were performed before statistical testing. According to the results of the Shapiro-Wilk- and the Kolmogorov-Smirnov-Test for normality, either two-sample Student’s *t*-test or two-tailed Mann–Whitney-*U*-Test for two sample groups or ONEWAY ANOVA or the Kruskal-Wallis-Test for multiple group comparisons (followed by post-hoc Bonferroni or Mann–Whitney-*U*-Tests with Bonferroni correction) were performed. For NORT a Wilcoxon-Test was performed to test if the ratio differed significantly from 1. For Y maze a one-sample *t*-test was performed to test if the percentage of correct alternations was significantly above 50. A *p*-value <0.05 was considered statistically significant. *p < 0.05, **p < 0.01, ***p < 0.001 indicate significantly different performances. Error bars represent SEM.

## Results

### Reduced β-amyloid pathology in PSAPP mice with a congenital deficiency of the adaptive immunity

**Fig. 1:**
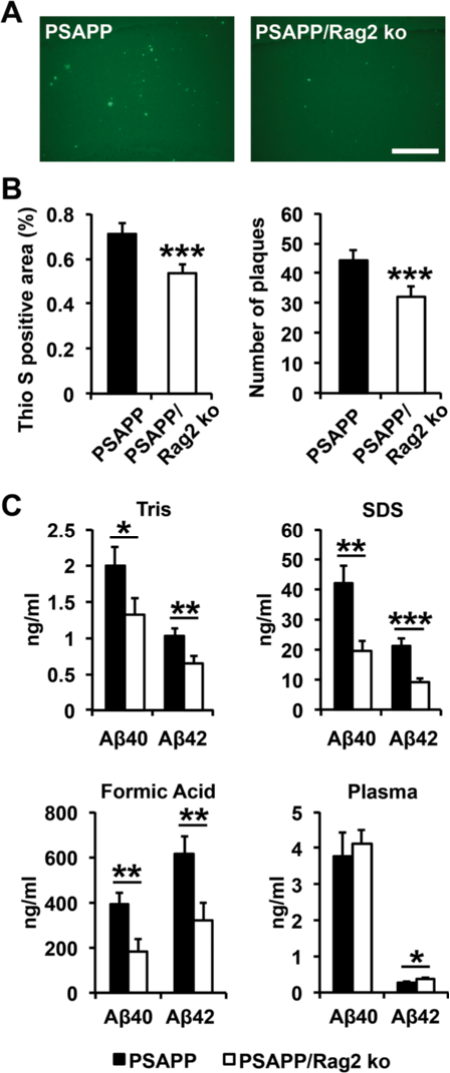
Reduced amyloid pathology and reduced Aβ levels in 8 month-old PSAPP/Rag2 ko mice in comparison to age-matched PSAPP mice. **a**, **b** Quantitative analysis of Thioflavin S stainings shows a 25 % reduction in amyloid load and amyloid plaque number in the B and T cell-ablated PSAPP/Rag2 ko mice as compared to the PSAPP mice with a functional adaptive immune system (representative images of cortical stainings are shown in (**a**)). Scale bar = 300 μm. ***p < 0.001. *n* = 13–15 per group. **c** Quantitative MSD analysis of Aβ40 and Aβ42 in plasma and cortical brain extracts shows a significant reduction in Tris soluble, SDS detergent soluble and formic acid soluble Aβ40 and Aβ42 levels in the PSAPP/Rag2 ko mice as compared to the PSAPP mice. The reduction in brain Aβ levels is accompanied by a significant increase in plasma Aβ42. *p < 0.05. **p < 0.01. ***p < 0.001. *n* = 9–14 per group

To investigate the effects of a specific congenital ablation of functional B and T cells on Aβ-related pathology *in vivo*, bigenic PSAPP mice expressing mouse/human amyloid precursor protein (Mo/HuAPP695swe) and mutant presenilin 1 (PS1-dE9) under the control of the mouse prion protein (PrP) promoter were crossed with the Rag2 ko mice. B and T cell-ablated PSAPP/Rag2 ko mice were analyzed together with immunocompetent PSAPP transgenic mice, non-transgenic Rag2 ko mice and WT control mice at two different ages. As expected, FACS analysis of whole blood confirmed the absence of functional lymphocytes in the two Rag2 ko mouse lines, Rag2 ko and PSAPP/Rag2 ko (Additional file 1: Figure S1). Notably, B and T cell numbers were similar between WT and the PSAPP transgenic mice, and no significant differences were observed in the systemic myeloid compartment among the four genotypes (Additional file 1: Figure S1). Histological analysis at 3 months of age (pre-plaque stage) showed no detectable amyloid plaque deposition in the Rag2-deficient PSAPP/Rag2 ko mice, nor in age-matched PSAPP mice; quantitative MSD analysis of cortical brain extracts and plasma revealed similar Aβ levels in the two genotypes at 3 months (Additional file 2: Figure 2). In contrast, at 8 months of age, Thioflavin-S stainings for fibrillar Aβ deposits (Fig. 1a, b) and immunostainings with the monoclonal anti-Aβ antibody 6E10 (data not shown) demonstrated abundant amyloid plaque pathology in the PSAPP mice which was significantly reduced in age-matched PSAPP/Rag2 ko mice. The 25–30 % reduction in amyloid plaque load and Aβ plaque numbers in the B and T cell-deficient PSAPP/Rag2 ko mice was accompanied by a marked decrease in soluble and insoluble brain Aβ40 and Aβ42 levels and paralleled by a mild increase in plasma Aβ42 in comparison to the PSAPP mice (Fig. 1a–c). These effects on Aβ pathology were associated neither with changes in APP expression nor with changes in APP processing, as revealed by Western blot analyses, suggesting that the reduction in Aβ load and Aβ levels was unlikely to be mediated by altered Aβ production (Additional file 3: Figure S3).

### Glial responses and cognitive behavior in Rag2-deficient PSAPP mice

**Fig. 2:**
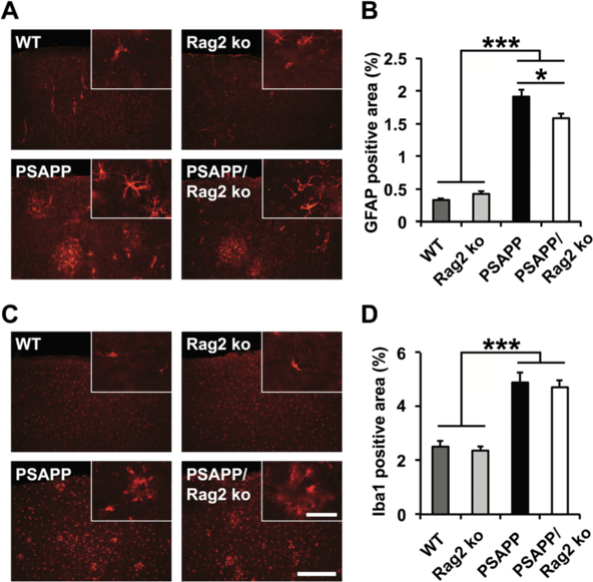
Glial responses in the absence of functional B and T cells at 8 months. **a**, **b** GFAP immunohistological analysis for astrocytes and (**c**, **d**) Iba1 immunostaining for microglia reveal significant increases in microgliosis in the amyloid-depositing APP transgenic mice (PSAPP/Rag2 ko and PSAPP) in comparison to the non-transgenic Rag2 ko and WT mice. GFAP immunoreactivity (**b**) – but not Iba1 immunoreactivity (**d**) – is significantly reduced in the PSAPP/Rag2 ko mice as compared to the PSAPP mice (representative images of cortical stainings are shown on the left). Scale bar = 300 μm (Insert: Scale bar = 40 μm). *p < 0.05. ***p < 0.001. *n* = 10–14 per group

To assess whether the reduction in Aβ pathology in 8 month-old PSAPP/Rag2 ko mice was associated with changes in glial cell responses, GFAP immunostainings for astrocytes and Iba1 stainings for microglia were performed. No significant differences in astroglia and microglial cells were observed among the four genotypes at the age of 3 months (Additional file 4: Figure S4). In 8 month-old animals, as anticipated, the degree of astrogliosis and microgliosis was significantly increased in the plaque-bearing PSAPP and PSAPP/Rag2 ko mice as compared to WT and Rag2 ko mice (Fig. 2a–d). The observed decrease in amyloid pathology in the PSAPP/Rag2 ko mice was accompanied by a significantly reduced GFAP immunoreactivity in comparison to the PSAPP mice (Fig. 2a, b). In contrast, Iba1 immunoreactivity appeared similar between the PSAPP/Rag2 ko and the PSAPP mice, suggesting a relative increase in β-amyloid-related microgliosis in the absence of functional B and T cells (Fig. 2c, d).

**Fig. 3:**
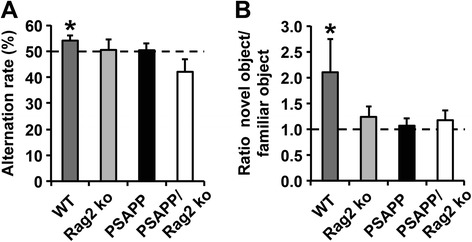
Impaired hippocampal memory performance in the PSAPP transgenic mice and both Rag2 ko-deficient mouse lines in comparison to the WT control. **a** Y maze performance was evaluated by correct alternations in %. **b** Recognition memory in the novel object recognition task (NORT) was measured by comparing the time spent with the novel and the familiar object. In the Y maze and the novel object recognition task performance was compared to chance level (50 % and 1, respectively). Significant differences are shown. *p < 0.05. *n* = 9–21 per group

To test whether B and T cell ablation and the observed effects on Aβ pathology in the PSAPP/Rag2 ko had any influence on cognitive outcome measures, we analyzed hippocampal-dependent cognitive performance in the four genotypes (WT, PSAPP, Rag2 ko and PSAPP/Rag2 ko). Spatial working memory was assessed in the Y maze, while recognition memory was tested in the Novel Object Recognition Task (NORT). At 8 months of age, PSAPP transgenic mice were characterized by significant impairments on both cognitive tasks in comparison to age-matched WT control mice (Fig. 3a, b). In line with previous reports showing reduced cognitive performance in mice lacking functional adaptive immune cells [48–50], similar results were observed for both immunocompromised mouse lines (Rag2 ko and PSAPP/Rag2 ko) independent of the APP transgene expression (Fig. 3a, b).

### Reduced amyloid pathology in PSAPP mice with an acquired deficiency of the adaptive immune system

**Fig. 4:**
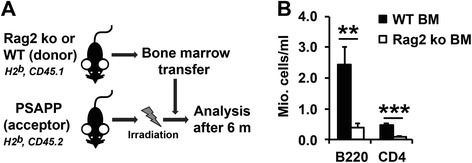
Bone marrow (BM) transfer experiment. **a** 12 month-old PSAPP mice (immunophenotype H2^b^, CD45.2) were lethally irradiated with two doses of 600 rads, then reconstituted with 5 x 10^6^ BM cells from either Rag2 ko or WT donor mice (immunophenotype H2^b^, CD45.1). **b** 6 months after transplantation, Rag2 ko BM recipients showed a fivefold reduction in B cells and CD4+ T cells, as determined by FACS staining of CD45+ blood leukocytes with antibodies against CD4 and the pan B-cell marker B220. **p < 0.01. ***p < 0.001 . *n* = 9–10 per group

**Fig. 5:**
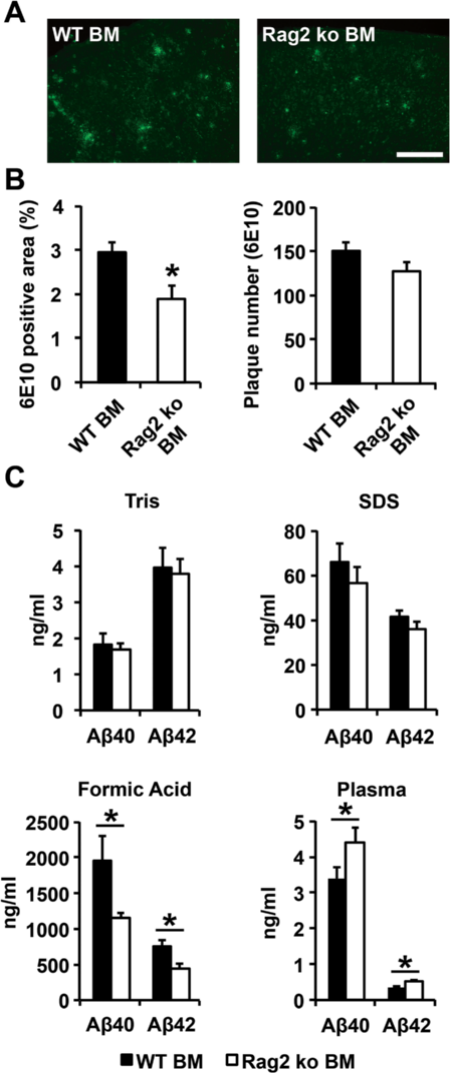
Reduced Aβ load in PSAPP mice reconstituted with Rag2 ko BM **a**, **b**). Quantitative analysis of free-floating sections stained with the monoclonal anti-Aβ antibody 6E10 shows a 35 % reduction in the Aβ-immunoreactivity, but no significant change in Aβ-immunoreactive plaque number, in Rag2 ko BM-transferred PSAPP mice as compared to WT BM-transferred PSAPP mice 6 months after reconstitution (at 18 months of age; representative images of cortical stainings are shown in (**a**)). Scale bar = 300 μm. *p < 0.05. *n* = 8–10 per group. (**c**) Quantitative MSD analysis of plasma and cortical brain extracts reveals a significant reduction in formic acid soluble Aβ40 and Aβ42 along with an increase in plasma Aβ levels in PSAPP mice transferred with Rag2 ko BM in comparison with WT BM-transferred PSAPP mice. *p < 0.05. *n* = 9–10 per group

Building on our results in the PSAPP/Rag2 ko mice lacking functional B and T cells from birth, we next sought to determine the effects of an acquired deficiency of the adaptive immunity on established Aβ-related pathology in the PSAPP model of β-amyloidosis. To achieve this goal, bone marrow (BM) chimeric mice were generated by adoptive transfer of BM cells from Rag2 ko and WT control mice into 12 month-old lethally irradiated PSAPP mice (Fig. 4a). FACS analysis of whole blood 6 months after BM transfer showed a fivefold reduction of mature B (B220+) and T (CD4+) lymphocytes in the Rag2 ko BM-reconstituted PSAPP mice, thus confirming the successful reduction of adaptive immune cells in this model (Fig. 4b). Histopathological analysis of Thioflavin-S-stained brain sections from 18 month-old mice revealed a similar amyloid burden between the WT BM-grafted PSAPP mice and the Rag2 ko BM-transferred PSAPP mice, suggesting that reconstitution with B and T cell-deficient BM had no significant impact on compact fibrillar Aβ deposits in this model (Additional file 5: Figure S5 A, B). Immunostainings with the monoclonal antibody 6E10, however, which recognizes a wide range of Aβ aggregates including diffuse Aβ deposits, showed a significantly reduced (around 35 %) Aβ plaque load, but no change in Aβ plaque numbers in the Rag2 ko BM-transferred PSAPP mice in comparison to WT BM-reconstituted mice (Fig. 5a, b). This reduction in 6E10-stained Aβ load was associated with a significant decrease in detergent insoluble (FA soluble) cortical brain Aβ40 and Aβ42 levels and a parallel increase in plasma Aβ40 and Aβ42 (Fig. 5c).

### Increased microgliosis and enhanced Aβ phagocytosis in PSAPP mice reconstituted with Rag2 ko BM

Similar to our findings in the crossbred mice, the reduction in Aβ pathology in the Rag2 ko BM-reconstituted PSAPP mice was not associated with changes in levels of full-length APP nor in changes of APP C-terminal fragments, again suggesting that the acquired deficiency in functional B and T cells did not influence Aβ levels by affecting APP metabolism (Additional file 6: Figure S6).

**Fig. 6:**
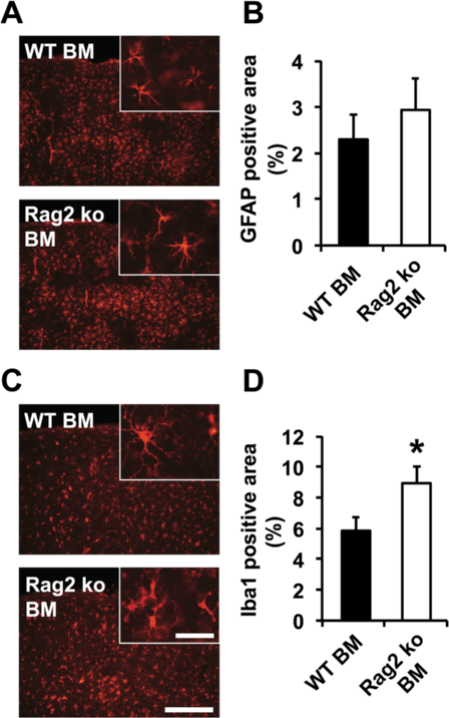
Glial responses in PSAPP mice reconstituted with WT and Rag2 ko BM. **a** Astrogliosis is unchanged in Rag2 ko BM-transferred mice in comparison to WT BM-transferred PSAPP mice 6 months after reconstitution (at 18 months; representative images of cortical stainings are shown on the left). **b** Quantitative analysis of GFAP immunostainings. **c**, **d** Iba1 immunohistological analysis reveals a significant increase in microgliosis in Rag2 ko BM-recipient PSAPP mice in comparison to WT BM-transferred PSAPP mice. Scale bar = 300 μm (Insert: Scale bar = 40 μm). *p < 0.05. *n* = 8–10 per group

**Fig. 7:**
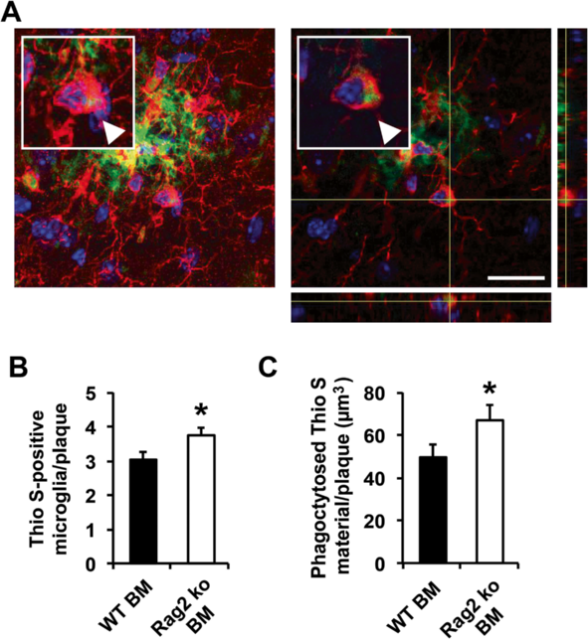
Increased number of Aβ-phagocytosing microglia in the Rag2 ko BM-reconstituted PSAPP mice. **a** Representative maximum projection confocal image (left panel) of a plaque-associated microglial cell containing Thioflavin S-positive Aβ material (arrowhead). Image shows microglia (Iba1, red) and amyloid deposits (Thioflavin S, green). Nuclei were counterstained by DAPI (blue). Orthogonal views of z-stack images are shown in the right panel. Scale bar = 20 μm (**b**) significantly increased number of amyloid plaque-associated Iba1-positive microglial cells containing Thioflavin S-positive Aβ material in the Rag2 ko BM-recipient PSAPP mice. *p < 0.05. n = 9 mice per group (on average 12 plaques per mouse analyzed) (**c**) significantly increased total volume of phagocytosed Aβ material in the Rag2 ko BM-reconstituted PSAPP mice in comparison to the WT BM-recipient control mice. *p < 0.05. n = 9 mice per group (on average 12 plaques per mouse analyzed)

To determine whether the reduction in Aβ pathology in the PSAPP mice reconstituted with Rag2 ko BM was associated with altered cellular responses of the brain’s innate immune system, GFAP and Iba1 immunostainings were performed 6 months after BM reconstitution (at 18 months of age). Quantitative immunostochemical analysis showed similar astrogliosis between WT and Rag2 ko BM-transferred PSAPP mice (Fig. 6a, b), but a significant increase in microglial cells in the Rag2 ko BM-recipient mice (Fig. 6c, d). Confocal microscope analysis of Iba1-positive cells decorating Thioflavin S-stained amyloid plaques, which were matched for size, revealed a trend towards elevated average numbers of plaque-associated microglia in the Rag2 ko BM-transferred PSAPP mice in comparison to the WT BM-recipient mice (Additional file 7: Figure S7 A, B). Importantly, both the number of Aβ-phagocytosing microglia and the total volume of the phagocytosed Thioflavin S-positive material per plaque were significantly increased in the PSAPP mice reconstituted with Rag2 ko BM, suggesting enhanced Aβ phagocytosis in the absence of functional T and B cells (Fig. 7a, b).

## Discussion

The ageing process is a major risk factor for sporadic Alzheimer’s disease and impacts negatively on an individual’s capacity to respond to immune challenges, a condition termed immunosenescence [51]. Human immunosenescence is accompanied by a deterioration of B and T cell-mediated adaptive immune responses, while innate immunity is rather preserved or even upregulated [51]. An even more pronounced reduction in systemic T and B cells has previously been reported by some authors in patients with Alzheimer’s disease [31], suggesting a “premature immunosenescence” phenotype specifically affecting adaptive immunity in the pathogenesis of the disease [52]. In the present study, we applied two different experimental paradigms to investigate the contribution of the adaptive immune system to cerebral β-amyloid pathology in an established APP transgenic mouse model of Alzheimer’s disease. The consequences of a congenital deficiency of the adaptive immunity were assessed in the PSAPP/Rag2 ko crossbred mice which were compared to immunocompetent PSAPP mice. To confirm the absence of T and B cells in this model and exclude potential effects of APP/mutant presenilin 1 on systemic immune compartments in the PSAPP mice [53], FACS analyses of whole blood as well as other peripheral immune organs (data not shown) were performed. Notably, no significant alterations of peripheral lymphocytes and myeloid cells were observed between PSAPP transgenic and WT control mice, thus excluding major systemic immune alterations in this model. Histological and biochemical analyses revealed a reduced β-amyloid pathology along with decreased brain Aβ levels in aged PSAPP/Rag2 ko mice in comparison to the PSAPP mice after onset of amyloid plaque deposition. This decrease in Aβ pathology was associated with an increase in plasma Aβ levels and a relative increase in Iba1-positive microglial cells, which was not observed in 3 month-old pre-plaque PSAPP/Rag2 ko mice, suggesting that the reduction in brain Aβ in the absence of functional B and T cells only occurred in the presence of Aβ plaque deposits and was likely mediated by amyloid-reactive microglia. In line with these findings, similar results were obtained in our second model system of an acquired adaptive immune deficiency: aged PSAPP mice reconstituted with Rag2 ko BM cells showed a reduced β-amyloid load, decreased brain Aβ and elevated plasma Aβ levels along with increased microgliosis and elevated numbers of plaque-associated Aβ-phagocytosing microglia. In contrast to our findings in the PSAPP/Rag2 ko crossbred mice, reconstitution of PSAPP transgenic mice with Rag2 ko BM resulted in milder effects on Aβ pathology, as revealed by a reduced 6E10-stained Aβ load, but no change in Thioflavin S-stained compact fibrillar Aβ deposits, decreased brain Aβ levels exclusively in the detergent insoluble protein fraction and similar levels of β-amyloid-associated astrogliosis as compared to the WT BM-recipient PSAPP controls. In our opinion, the observed milder effects on Aβ pathology may at least partly be explained by the incomplete ablation of T and B cells in the BM transfer paradigm (versus a complete ablation of lymphocytes in the crossbreeding approach). Moreover, additional aspects, e.g. the older age of PSAPP transgenic mice in the BM transfer experiment and/or shorter follow-up periods (6 months versus 8 months in the crossbreeding approach) have to be taken into account.

In our experimental set up Rag2 ko mice with a combined T and B cell deficiency were used. This approach allowed us to simulate the conditions of “premature immunosenescence” in a mouse model of cerebral β-amyloidosis and investigate the effects of a general adaptive immune deficiency on Aβ pathology. Based on this experimental design, however, we were not able to delineate a specific lymphocyte subpopulation accounting for the effects on microglia and cerebral amyloid deposition. Although we cannot exclude that a lack of B cell-mediated humoral immunity might have contributed to the observed effects, this possibility appears to be less likely in our view for several reasons. On the one hand, B cells, unlike T cells, have not been detectable in brain parenchyma of Alzheimer’s disease patients [54] nor in brains of PSAPP transgenic mice, as revealed by immunohistochemistry and brain FACS analysis (Ferretti et al., submitted). On the other hand, based on several previous studies suggesting a role for naturally occurring Aβ autoantibodies in restricting brain amyloidosis [12, 18–20], a lack of Aβ-reactive B cells would probably be expected to result in exacerbated rather than reduced amyloid pathology. In line with this notion, progressive age-related amyloid plaque deposition was previously associated with a specific reduction of naturally occurring serum Aβ autoantibodies in the Tg2576 APP transgenic mouse model of Alzheimer’s disease [55]. In contrast to B cells, T cells have been repeatedly detected as infiltrating brain tissue both in human Alzheimer’s disease brains and in animal models of the disease [24–29, 56, 57] Ferretti et al., submitted). Although the exact role of T lymphocytes in the disease process remains incompletely understood, both proinflammatory and neurotoxic as well as antiinflammatory and neurotrophic mechanisms have been attributed to their presence in brain parenchyma [58]. In certain rat and APP transgenic mouse models of Alzheimer’s disease, for example, specific effector CD4^+^ T cells secreting IFN-γ, IL-17 or IL-22 were linked to increased neuroinflammation and exacerbation of disease progression [59, 60]. In other animal studies, however, IFN-γ-dependent targeting of Aβ-specific Th1-polarized CD4^+^ cells to amyloid plaques was shown to reduce amyloid pathology without promoting toxic neuroinflammation [61, 62].

A role for the adaptive immune system in neuroinflammation and brain pathology has also been proposed in other (non-Alzheimer’s disease) neurodegenerative conditions. Removal of CD4^+^ T lymphocytes, but not CD8^+^ lymphocytes, for example, was previously associated with reduced MPTP-induced neuronal cell loss in a mouse model of Parkinson’s disease [63]. In contrast, the same T cell population was shown to provide neuroprotection in an animal model of familial amyotrophic lateral sclerosis (ALS) [64, 65]. In this work, mSOD1^G93A^/Rag2 ko mice and CD4^+^ T cell-ablated mSOD1^G93A^/CD4 ko mice showed accelerated disease progression and increased motoneuron degeneration, which was associated with increased mRNA levels for proinflammatory cytokines and NOX2, and decreased levels of trophic factors and glutamate transporters [64]. Interestingly, bone marrow reconstitution of mSOD1^G93A^/Rag2 ko mice with T cells led to a prolonged survival and suppressed cytotoxicity [64]. These beneficial effects were accompanied by the induction of an M2 protective microglial phenotype, which was mainly attributed to the presence of CD4^+^CD25^+^FoxP3^+^ regulatory T lymphocytes (Tregs) [65]. Suppressive effects on both the adaptive and the innate arm of the immune system have been well documented for Tregs [66–68]. Their lack in the Rag2-deficient PSAPP transgenic mice may therefore, at least in part, account for the observed increased neuroinflammatory phenotype and microglia-mediated phagocytosis of Aβ aggregrates. This possibility is further supported by recent findings in the 5XFAD AD mouse model of AD, in which transient depletion of Foxp3^+^ Tregs, or pharmacological inhibition of their activity, was similarly shown to mitigate amyloid plaque clearance [69]. Tregs secrete various anti-inflammatory cytokines (such as IL-4, IL-10 and IL-13) and have been shown to promote alternatively activated (M2) responses and attenuate proinflammatory (M1) microglial responses [68, 70, 71]. Notably, microglial polarization towards the M1 response has been associated with reduced amyloid pathology [72, 73], which would also be in line with our findings in the T and B cell-ablated PSAPP mice. Despite significantly reduced Aβ levels, an increased proinflammatory phenotype in the absence of functional adaptive immune cells may be a double-edged sword. In fact, proinflammatory microglial responses have also been associated with detrimental consequences such as increased tau pathology and exacerbated neurotoxicity [73]. In our study, the beneficial effects on Aβ pathology were not accompanied by a reversal of hippocampal-dependent memory deficits in the PSAPP/Rag2 ko mice. Non-transgenic Rag2 ko mice, however, were equally impaired on two different hippocampal-dependent tasks in comparison to the WT control, suggesting additional Aβ-independent effects of the T and B cell ablation on cognitive performance. In line with these findings, a properly functioning adaptive immune system has previously been shown to be involved in maintaining cognitive health and hippocampal memory formation in several immunocomprised mouse models lacking functional T cells [48–50]. These effects were proposed to be mainly mediated by non-CNS specific CD4^+^ T cells playing a role in brain plasticity and promoting neurogenesis in a BDNF-dependent manner in the adult hippocampus [48–50].

## Conclusions

In summary, the results of our current study demonstrate an impact of the adaptive immunity on Alzheimer’s disease-related β-amyloid pathology in vivo and suggest a crosstalk between the adaptive immune system and microglial cells as a likely underlying mechanism. In two independent experimental approaches, ablation of functional T and B cells led to reduced brain Aβ pathology and decreased Aβ levels along with increased microgliosis and clearance of Aβ aggregates. Ablation of the adaptive immune system, however, did not influence Aβ levels and glial cells in younger APP transgenic mice at a pre-plaque stage suggesting that the modulation of microglial responses to Aβ is specifically related to the presence of amyloid deposits. Taken together, our findings point to a yet unidentified role of the adaptive immunity in modulating and dampening microglial responses to misfolded Aβ peptides in the brain. From an evolutionary perspective, this could be a protective compensatory mechanism of the adaptive immune system aimed to control or reduce potentially toxic neuroinflammatory responses to Aβ aggregates, even though at the cost of an increased amyloid plaque deposition. Future studies will be needed to delineate the specific lymphocyte subpopulation(s) and major immune mediators involved in these processes. A better understanding of the underlying neuroimmunological mechanisms may ultimately pave the way for more effective and safer therapies harnessing adaptive immunity to treat cerebral β-amyloidosis.

## Additional files

Additional file 1: Figure S1. Absence of lymphocytes in both Rag2 ko mouse lines. 8 month-old Rag2 ko and PSAPP/Rag2 ko mice show virtually no B cells and CD4+ T cells, whereas age-matched WT and PSAPP transgenic animals show comparable numbers of B and CD4+ T cells as determined by FACS staining of CD45+ blood lymphocytes with antibodies against CD4 and the pan B-cell marker B220. In addition, no significant differences in the myeloid monocytic compartment (Ly6C^high^ and Ly6C^intermediate^) are observed among the four genotypes. ^#^ *p* = 0.05-0.1. **p* < 0.05. *n* = 4–6 per group. (TIFF 41631 kb) Additional file 2: Figure S2. Lack of amyloid plaque deposition at 3 months. (**A**) Thioflavin-S staining confirms absence of amyloid plaque deposition in 3 month-old PSAPP and age-matched PSAPP/Rag2 ko mice (representative images of cortical stainings are shown). (**B**) Quantitative MSD analysis of Aβ40 and Aβ42 in plasma and cortical tissue homogenized using sequential extraction to obtain different protein fractions: Tris soluble; SDS detergent soluble; formic acid soluble fraction (**B**). Statistical analysis reveals no differences in Aβ levels between PSAPP/Rag2 ko mice and the PSAPP mice at 3 months. Scale bar = 300 μm. *n* = 14–15 per group. (TIF 26755 kb) Additional file 3: Figure S3. No difference in APP processing at 8 months. (**A**) Western blot analysis of cortical SDS extracts from 8 month-old mice in comparison to age-matched PSAPP mice. Values were normalized to GAPDH. (**B**) Quantification shows no significant differences in full length APP and APP C-terminal fragments (C99 and C83) between PSAPP/Rag2 ko and PSAPP mice. *n* = 7 per group. (TIF 29174 kb) Additional file 4: Figure S4. No difference in gliosis at 3 months of age (**A**, **B**) GFAP immunohistological analysis for astrocytes and (**C**, **D**) Iba1 immunostaining for microglia (representative images of cortical stainings are shown on the left). Statistical analysis reveals no significant differences in gliosis between the four genotypes at 3 months of age. Scale bar = 300 μm (Insert: Scale bar = 40 μm). *n* = 11–13 per group. (TIFF 158644 kb) Additional file 5: Figure S5. No difference in Thioflavin S-stained amyloid deposits between Rag2 ko and WT BM-transferred PSAPP mice. (**A**) Representative images of Thioflavin S-stained cortical amyloid deposits. (**B**) Quantitative analysis of Thioflavin S stainings 6 months after transplantation (at the age of 18 months) shows no difference in amyloid load and amyloid plaque number. Scale bar = 300 μm. *n* = 8–10 per group. (TIF 16319 kb) Additional file 6: Figure S6. No difference in APP processing. (**A**) Western blot analysis of cortical SDS extracts from 18-month-old PSAPP mice reconstituted with WT or Rag2 ko BM 6 months before sacrifice. Values were normalized to GAPDH. (**B**) Quantification reveals no significant differences in full length APP and APP C-terminal fragments (C99 and C83). *n* = 6 per group. (TIF 26355 kb) Additional file 7: Figure S7. Similar size of amyloid plaques analyzed by confocal microscopy. (**A**) Comparable average size of analyzed amyloid plaques between WT and Rag2 ko BM-reconstituted PSAPP mice at the age of 18 months. On average 12 Thioflavin S-stained amyloid plaques per mouse (*n* = 9 mice per group) were analyzed by laser scanning confocal microscopy. *p* = 0.711. (**B**) Trend towards an increased average number of Iba1-positive cells per plaque in the Rag2 ko BM-recipient PSAPP mice in comparison to the WT BM-reconstituted PSAPP mice at 18 months. Again, on average 12 Thioflavin S-stained amyloid plaques per mouse (*n* = 9 mice per group) were analyzed by laser scanning confocal microscopy. ^#^ *p* = 0.086. (TIF 8887 kb)

## References

[1] Amor S, Peferoen LA, Vogel DY, Breur M, van der Valk P, Baker D (2014). Inflammation in neurodegenerative diseases--an update. Immunology.

[2] Querfurth HW, LaFerla FM (2010). Alzheimer’s disease. N Engl J Med.

[3] Selkoe DJ (2011) Alzheimer's disease. Cold Spring Harb Perspect Biol 3: doi:10.1101/cshperspect.a004457. 10.1101/cshperspect.a004457 PMC3119915 21576255

[4] Akiyama H, Barger S, Barnum S, Bradt B, Bauer J, Cole GM (2000). Inflammation and Alzheimer’s disease. Neurobiol Aging.

[5] Heneka MT, Carson MJ, El Khoury J, Landreth GE, Brosseron F, Feinstein DL (2015). Neuroinflammation in Alzheimer’s disease. Lancet Neurol.

[6] Heppner FL, Ransohoff RM, Becher B (2015). Immune attack: the role of inflammation in Alzheimer disease. Nat Rev Neurosci.

[7] Wyss-Coray T (2006). Inflammation in Alzheimer disease: driving force, bystander or beneficial response?. Nat Med.

[8] Lucin KM, Wyss-Coray T (2009). Immune activation in brain aging and neurodegeneration: too much or too little?. Neuron.

[9] Heneka MT, Golenbock DT, Latz E (2015). Innate immunity in Alzheimer's disease. Nat Immunol.

[10] Du Y, Dodel R, Hampel H, Buerger K, Lin S, Eastwood B (2001). Reduced levels of amyloid beta-peptide antibody in Alzheimer disease. Neurology.

[11] Geylis V, Kourilov V, Meiner Z, Nennesmo I, Bogdanovic N, Steinitz M (2005). Human monoclonal antibodies against amyloid-beta from healthy adults. Neurobiol Aging.

[12] Kellner A, Matschke J, Bernreuther C, Moch H, Ferrer I, Glatzel M (2009). Autoantibodies against beta-amyloid are common in Alzheimer's disease and help control plaque burden. Ann Neurol.

[13] Maftei M, Thurm F, Schnack C, Tumani H, Otto M, Elbert T (2013). Increased levels of antigen-bound beta-amyloid autoantibodies in serum and cerebrospinal fluid of Alzheimer's disease patients. PLoS One.

[14] Moir RD, Tseitlin KA, Soscia S, Hyman BT, Irizarry MC, Tanzi RE (2005). Autoantibodies to redox-modified oligomeric Abeta are attenuated in the plasma of Alzheimer's disease patients. J Biol Chem.

[15] Mruthinti S, Buccafusco JJ, Hill WD, Waller JL, Jackson TW, Zamrini EY (2004). Autoimmunity in Alzheimer's disease: increased levels of circulating IgGs binding Abeta and RAGE peptides. Neurobiol Aging.

[16] Qu BX, Gong Y, Moore C, Fu M, German DC, Chang LY (2014). Beta-amyloid auto-antibodies are reduced in Alzheimer’s disease. J Neuroimmunol.

[17] Weksler ME, Relkin N, Turkenich R, LaRusse S, Zhou L, Szabo P (2002). Patients with Alzheimer disease have lower levels of serum anti-amyloid peptide antibodies than healthy elderly individuals. Exp Gerontol.

[18] Gold M, Mengel D, Roskam S, Dodel R, Bach JP (2013). Mechanisms of action of naturally occurring antibodies against beta-amyloid on microglia. J Neuroinflammation.

[19] Neff F, Wei X, Nolker C, Bacher M, Du Y, Dodel R (2008). Immunotherapy and naturally occurring autoantibodies in neurodegenerative disorders. Autoimmun Rev.

[20] Szabo P, Relkin N, Weksler ME (2008). Natural human antibodies to amyloid beta peptide. Autoimmun Rev.

[21] Britschgi M, Olin CE, Johns HT, Takeda-Uchimura Y, LeMieux MC, Rufibach K (2009). Neuroprotective natural antibodies to assemblies of amyloidogenic peptides decrease with normal aging and advancing Alzheimer’s disease. Proc Natl Acad Sci U S A.

[22] Dodel R, Balakrishnan K, Keyvani K, Deuster O, Neff F, Andrei-Selmer LC (2011). Naturally occurring autoantibodies against beta-amyloid: investigating their role in transgenic animal and in vitro models of Alzheimer’s disease. J Neurosci.

[23] Mengel D, Roskam S, Neff F, Balakrishnan K, Deuster O, Gold M (2013). Naturally occurring autoantibodies interfere with beta-amyloid metabolism and improve cognition in a transgenic mouse model of Alzheimer's disease 24 h after single treatment. Transl Psychiatry.

[24] Itagaki S, McGeer PL, Akiyama H (1988). Presence of T-cytotoxic suppressor and leucocyte common antigen positive cells in Alzheimer’s disease brain tissue. Neurosci Lett.

[25] McGeer PL, Akiyama H, Itagaki S, McGeer EG (1989). Immune system response in Alzheimer's disease. Can J Neurol Sci.

[26] Parachikova A, Agadjanyan MG, Cribbs DH, Blurton-Jones M, Perreau V, Rogers J (2007). Inflammatory changes parallel the early stages of Alzheimer disease. Neurobiol Aging.

[27] Pirttila T, Mattinen S, Frey H (1992). The decrease of CD8-positive lymphocytes in Alzheimer's disease. J Neurol Sci.

[28] Rogers J, Rovigatti U (1988). Immunologic and tissue culture approaches to the neurobiology of aging. Neurobiol Aging.

[29] Togo T, Akiyama H, Iseki E, Kondo H, Ikeda K, Kato M (2002). Occurrence of T cells in the brain of Alzheimer’s disease and other neurological diseases. J Neuroimmunol.

[30] Monsonego A, Zota V, Karni A, Krieger JI, Bar-Or A, Bitan G (2003). Increased T cell reactivity to amyloid beta protein in older humans and patients with Alzheimer disease. J Clin Invest.

[31] Richartz-Salzburger E, Batra A, Stransky E, Laske C, Kohler N, Bartels M (2007). Altered lymphocyte distribution in Alzheimer’s disease. J Psychiatr Res.

[32] Jozwik A, Landowski J, Bidzan L, Fulop T, Bryl E, Witkowski JM (2012). Beta-amyloid peptides enhance the proliferative response of activated CD4CD28 lymphocytes from Alzheimer disease patients and from healthy elderly. PLoS One.

[33] Lueg G, Gross CC, Lohmann H, Johnen A, Kemmling A, Deppe M (2015). Clinical relevance of specific T-cell activation in the blood and cerebrospinal fluid of patients with mild Alzheimer's disease. Neurobiol Aging.

[34] Pellicano M, Larbi A, Goldeck D, Colonna-Romano G, Buffa S, Bulati M (2012). Immune profiling of Alzheimer patients. J Neuroimmunol.

[35] Speciale L, Calabrese E, Saresella M, Tinelli C, Mariani C, Sanvito L (2007). Lymphocyte subset patterns and cytokine production in Alzheimer’s disease patients. Neurobiol Aging.

[36] Jankowsky JL, Fadale DJ, Anderson J, Xu GM, Gonzales V, Jenkins NA (2004). Mutant presenilins specifically elevate the levels of the 42 residue beta-amyloid peptide in vivo: evidence for augmentation of a 42-specific gamma secretase. Hum Mol Genet.

[37] Shinkai Y, Rathbun G, Lam KP, Oltz EM, Stewart V, Mendelsohn M (1992). RAG-2-deficient mice lack mature lymphocytes owing to inability to initiate V(D)J rearrangement. Cell.

[38] Jankowsky JL, Melnikova T, Fadale DJ, Xu GM, Slunt HH, Gonzales V (2005). Environmental enrichment mitigates cognitive deficits in a mouse model of Alzheimer’s disease. J Neurosci.

[39] Kilkenny C, Browne WJ, Cuthill IC, Emerson M, Altman DG (2010). Improving bioscience research reporting: The ARRIVE guidelines for reporting animal research. J Pharmacol Pharmacother.

[40] Kulic L, McAfoose J, Welt T, Tackenberg C, Spani C, Wirth F (2012). Early accumulation of intracellular fibrillar oligomers and late congophilic amyloid angiopathy in mice expressing the Osaka intra-Abeta APP mutation. Transl Psychiatry.

[41] Paxinos G, Franklin KBJ (2012) The Mouse Brain in Stereotaxic Coordinates (4th Edition). Academic Press, City

[42] Knobloch M, Konietzko U, Krebs DC, Nitsch RM (2007). Intracellular Abeta and cognitive deficits precede beta-amyloid deposition in transgenic arcAbeta mice. Neurobiol Aging.

[43] Chakrabarty P, Li A, Ceballos-Diaz C, Eddy JA, Funk CC, Moore B (2015). IL-10 alters immunoproteostasis in APP mice, increasing plaque burden and worsening cognitive behavior. Neuron.

[44] Simen BB, Duman CH, Simen AA, Duman RS (2006). TNFalpha signaling in depression and anxiety: behavioral consequences of individual receptor targeting. Biol Psychiatry.

[45] Tang YP, Wang H, Feng R, Kyin M, Tsien JZ (2001). Differential effects of enrichment on learning and memory function in NR2B transgenic mice. Neuropharmacology.

[46] Holcomb L, Gordon MN, McGowan E, Yu X, Benkovic S, Jantzen P (1998). Accelerated Alzheimer-type phenotype in transgenic mice carrying both mutant amyloid precursor protein and presenilin 1 transgenes. Nat Med.

[47] Holcomb LA, Gordon MN, Jantzen P, Hsiao K, Duff K, Morgan D (1999). Behavioral changes in transgenic mice expressing both amyloid precursor protein and presenilin-1 mutations: lack of association with amyloid deposits. Behav Genet.

[48] Brynskikh A, Warren T, Zhu J, Kipnis J (2008). Adaptive immunity affects learning behavior in mice. Brain Behav Immun.

[49] Kipnis J, Cohen H, Cardon M, Ziv Y, Schwartz M (2004). T cell deficiency leads to cognitive dysfunction: implications for therapeutic vaccination for schizophrenia and other psychiatric conditions. Proc Natl Acad Sci U S A.

[50] Wolf SA, Steiner B, Akpinarli A, Kammertoens T, Nassenstein C, Braun A (2009). CD4-positive T lymphocytes provide a neuroimmunological link in the control of adult hippocampal neurogenesis. J Immunol.

[51] Martorana A, Bulati M, Buffa S, Pellicano M, Caruso C, Candore G (2012). Immunosenescence, inflammation and Alzheimer's disease. Longev Healthspan.

[52] Richartz-Salzburger E, Stransky E, Laske C, Kohler N (2010). Premature immunosenescence: a pathogenetic factor in Alzheimer’s disease?. Nervenarzt.

[53] Zhu Y, Obregon D, Hou H, Giunta B, Ehrhart J, Fernandez F (2011). Mutant presenilin-1 deregulated peripheral immunity exacerbates Alzheimer-like pathology. J Cell Mol Med.

[54] Rogers J, Luber-Narod J, Styren SD, Civin WH (1988). Expression of immune system-associated antigens by cells of the human central nervous system: relationship to the pathology of Alzheimer’s disease. Neurobiol Aging.

[55] Sohn JH, So JO, Kim H, Nam EJ, Ha HJ, Kim YH (2007). Reduced serum level of antibodies against amyloid beta peptide is associated with aging in Tg2576 mice. Biochem Biophys Res Commun.

[56] Monsonego A, Imitola J, Petrovic S, Zota V, Nemirovsky A, Baron R (2006). Abeta-induced meningoencephalitis is IFN-gamma-dependent and is associated with T cell-dependent clearance of Abeta in a mouse model of Alzheimer's disease. Proc Natl Acad Sci U S A.

[57] Town T, Tan J, Flavell RA, Mullan M (2005). T-cells in Alzheimer’s disease. Neuromolecular Med.

[58] Anderson KM, Olson KE, Estes KA, Flanagan K, Gendelman HE, Mosley RL (2014). Dual destructive and protective roles of adaptive immunity in neurodegenerative disorders. Transl Neurodegener.

[59] Browne TC, McQuillan K, McManus RM, O'Reilly JA, Mills KH, Lynch MA (2013). IFN-gamma Production by amyloid beta-specific Th1 cells promotes microglial activation and increases plaque burden in a mouse model of Alzheimer’s disease. J Immunol.

[60] Zhang J, Ke KF, Liu Z, Qiu YH, Peng YP (2013). Th17 cell-mediated neuroinflammation is involved in neurodegeneration of abeta1-42-induced Alzheimer’s disease model rats. PLoS One.

[61] Fisher Y, Nemirovsky A, Baron R, Monsonego A (2010). T cells specifically targeted to amyloid plaques enhance plaque clearance in a mouse model of Alzheimer’s disease. PLoS One.

[62] Fisher Y, Strominger I, Biton S, Nemirovsky A, Baron R, Monsonego A (2014). Th1 polarization of T cells injected into the cerebrospinal fluid induces brain immunosurveillance. J Immunol.

[63] Brochard V, Combadiere B, Prigent A, Laouar Y, Perrin A, Beray-Berthat V (2009). Infiltration of CD4+ lymphocytes into the brain contributes to neurodegeneration in a mouse model of Parkinson disease. J Clin Invest.

[64] Beers DR, Henkel JS, Zhao W, Wang J, Appel SH (2008). CD4+ T cells support glial neuroprotection, slow disease progression, and modify glial morphology in an animal model of inherited ALS. Proc Natl Acad Sci U S A.

[65] Beers DR, Henkel JS, Zhao W, Wang J, Huang A, Wen S (2011). Endogenous regulatory T lymphocytes ameliorate amyotrophic lateral sclerosis in mice and correlate with disease progression in patients with amyotrophic lateral sclerosis. Brain.

[66] Sakaguchi S (2005). Naturally arising Foxp3-expressing CD25 + CD4+ regulatory T cells in immunological tolerance to self and non-self. Nat Immunol.

[67] Sakaguchi S, Yamaguchi T, Nomura T, Ono M (2008). Regulatory T cells and immune tolerance. Cell.

[68] Tiemessen MM, Jagger AL, Evans HG, van Herwijnen MJ, John S, Taams LS (2007). CD4 + CD25 + Foxp3+ regulatory T cells induce alternative activation of human monocytes/macrophages. Proc Natl Acad Sci U S A.

[69] Baruch K, Rosenzweig N, Kertser A, Deczkowska A, Sharif AM, Spinrad A (2015). Breaking immune tolerance by targeting Foxp3(+) regulatory T cells mitigates Alzheimer's disease pathology. Nat Commun.

[70] Avidan H, Kipnis J, Butovsky O, Caspi RR, Schwartz M (2004). Vaccination with autoantigen protects against aggregated beta-amyloid and glutamate toxicity by controlling microglia: effect of CD4 + CD25+ T cells. Eur J Immunol.

[71] Reynolds AD, Stone DK, Hutter JA, Benner EJ, Mosley RL, Gendelman HE (2010). Regulatory T cells attenuate Th17 cell-mediated nigrostriatal dopaminergic neurodegeneration in a model of Parkinson's disease. J Immunol.

[72] Weekman EM, Sudduth TL, Abner EL, Popa GJ, Mendenhall MD, Brothers HM (2014). Transition from an M1 to a mixed neuroinflammatory phenotype increases amyloid deposition in APP/PS1 transgenic mice. J Neuroinflammation.

[73] Wilcock DM (2012). A changing perspective on the role of neuroinflammation in Alzheimer’s disease. Int J Alzheimers Dis.

