# Supplementary figures and images for: Reduced β-amyloid pathology in an APP transgenic mouse model of Alzheimer’s disease lacking functional B and T cells
Source: Acta Neuropathol Commun. 2015 Nov 11;3:71. doi: 10.1186/s40478-015-0251-x (PMC4642668; doi:10.1186/s40478-015-0251-x)

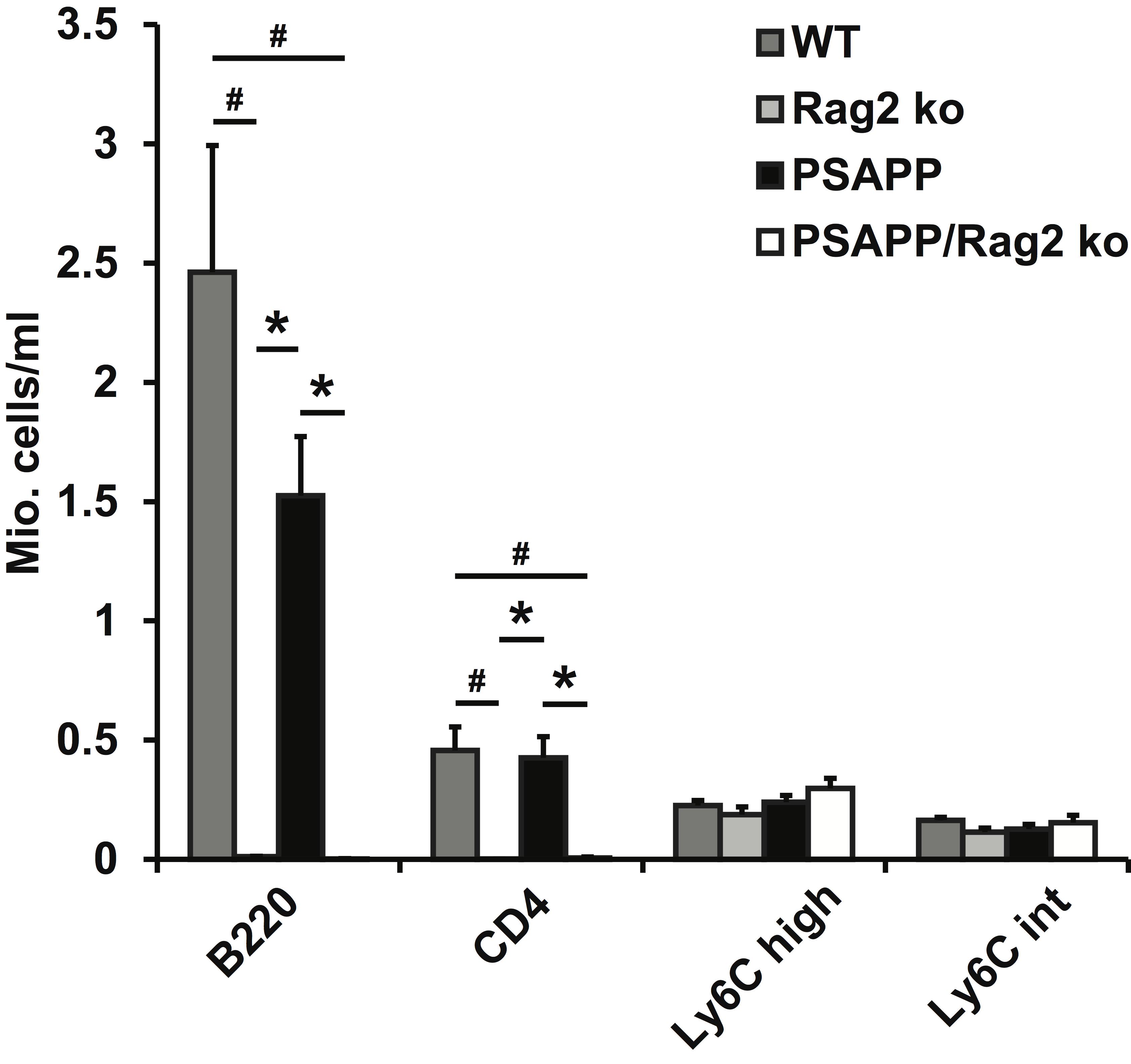

Supplement: Additional file 1: Figure S1. — Absence of lymphocytes in both Rag2 ko mouse lines. 8 month-old Rag2 ko and PSAPP/Rag2 ko mice show virtually no B cells and CD4+ T cells, whereas age-matched WT and PSAPP transgenic animals show comparable numbers of B and CD4+ T cells as determined by FACS staining of CD45+ blood lymphocytes with antibodies against CD4 and the pan B-cell marker B220. In addition, no significant differences in the myeloid monocytic compartment (Ly6Chigh and Ly6Cintermediate) are observed among the four genotypes. # p = 0.05-0.1. *p < 0.05. n = 4–6 per group. (TIFF 41631 kb) [file 40478_2015_251_MOESM1_ESM.tiff]

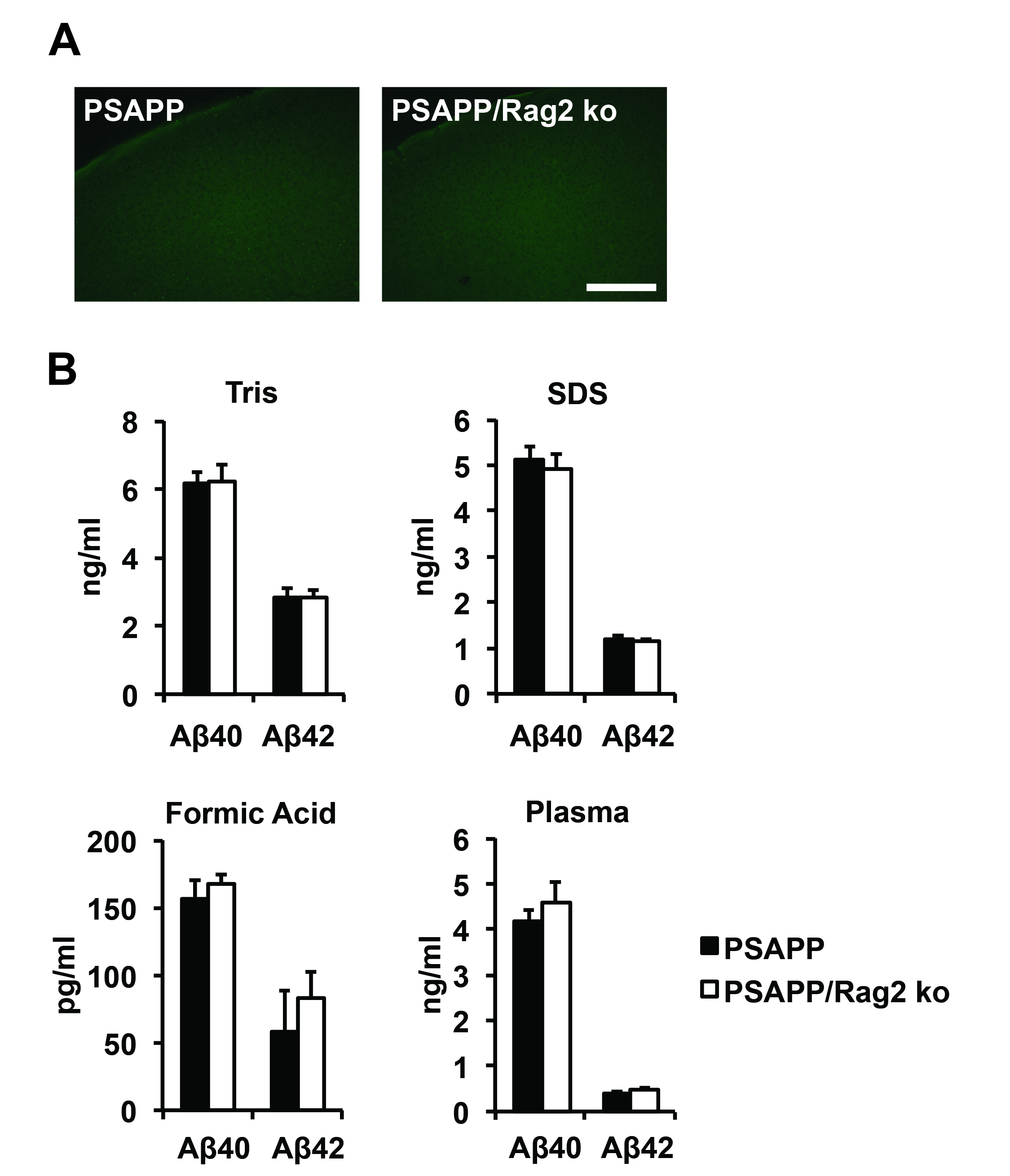

Supplement: Additional file 2: Figure S2. — Lack of amyloid plaque deposition at 3 months. (A) Thioflavin-S staining confirms absence of amyloid plaque deposition in 3 month-old PSAPP and age-matched PSAPP/Rag2 ko mice (representative images of cortical stainings are shown). (B) Quantitative MSD analysis of Aβ40 and Aβ42 in plasma and cortical tissue homogenized using sequential extraction to obtain different protein fractions: Tris soluble; SDS detergent soluble; formic acid soluble fraction (B). Statistical analysis reveals no differences in Aβ levels between PSAPP/Rag2 ko mice and the PSAPP mice at 3 months. Scale bar = 300 μm. n = 14–15 per group. (TIF 26755 kb) [file 40478_2015_251_MOESM2_ESM.tif]

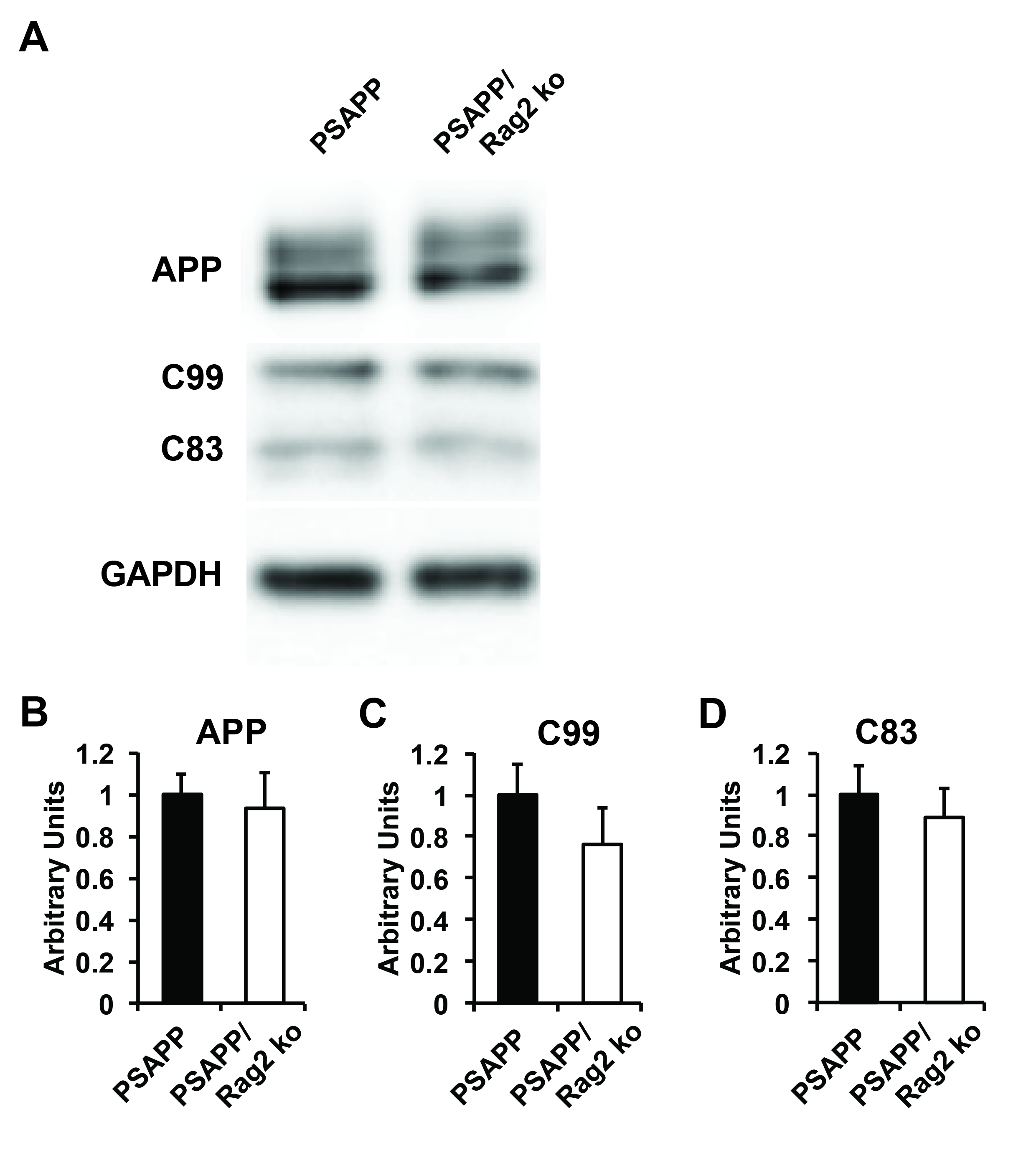

Supplement: Additional file 3: Figure S3. — No difference in APP processing at 8 months. (A) Western blot analysis of cortical SDS extracts from 8 month-old mice in comparison to age-matched PSAPP mice. Values were normalized to GAPDH. (B) Quantification shows no significant differences in full length APP and APP C-terminal fragments (C99 and C83) between PSAPP/Rag2 ko and PSAPP mice. n = 7 per group. (TIF 29174 kb) [file 40478_2015_251_MOESM3_ESM.tif]

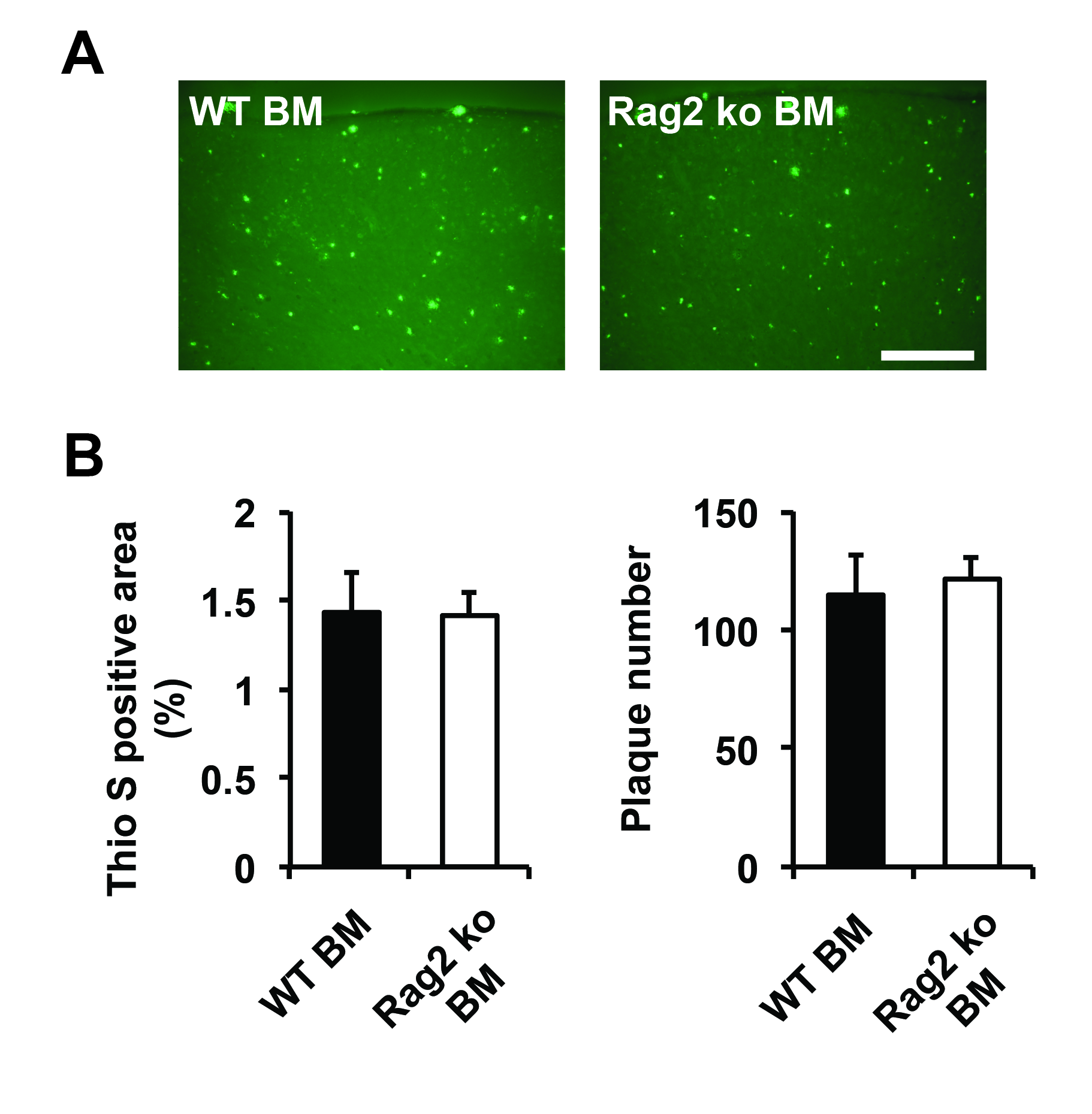

Supplement: Additional file 5: Figure S5. — No difference in Thioflavin S-stained amyloid deposits between Rag2 ko and WT BM-transferred PSAPP mice. (A) Representative images of Thioflavin S-stained cortical amyloid deposits. (B) Quantitative analysis of Thioflavin S stainings 6 months after transplantation (at the age of 18 months) shows no difference in amyloid load and amyloid plaque number. Scale bar = 300 μm. n = 8–10 per group. (TIF 16319 kb) [file 40478_2015_251_MOESM5_ESM.tif]

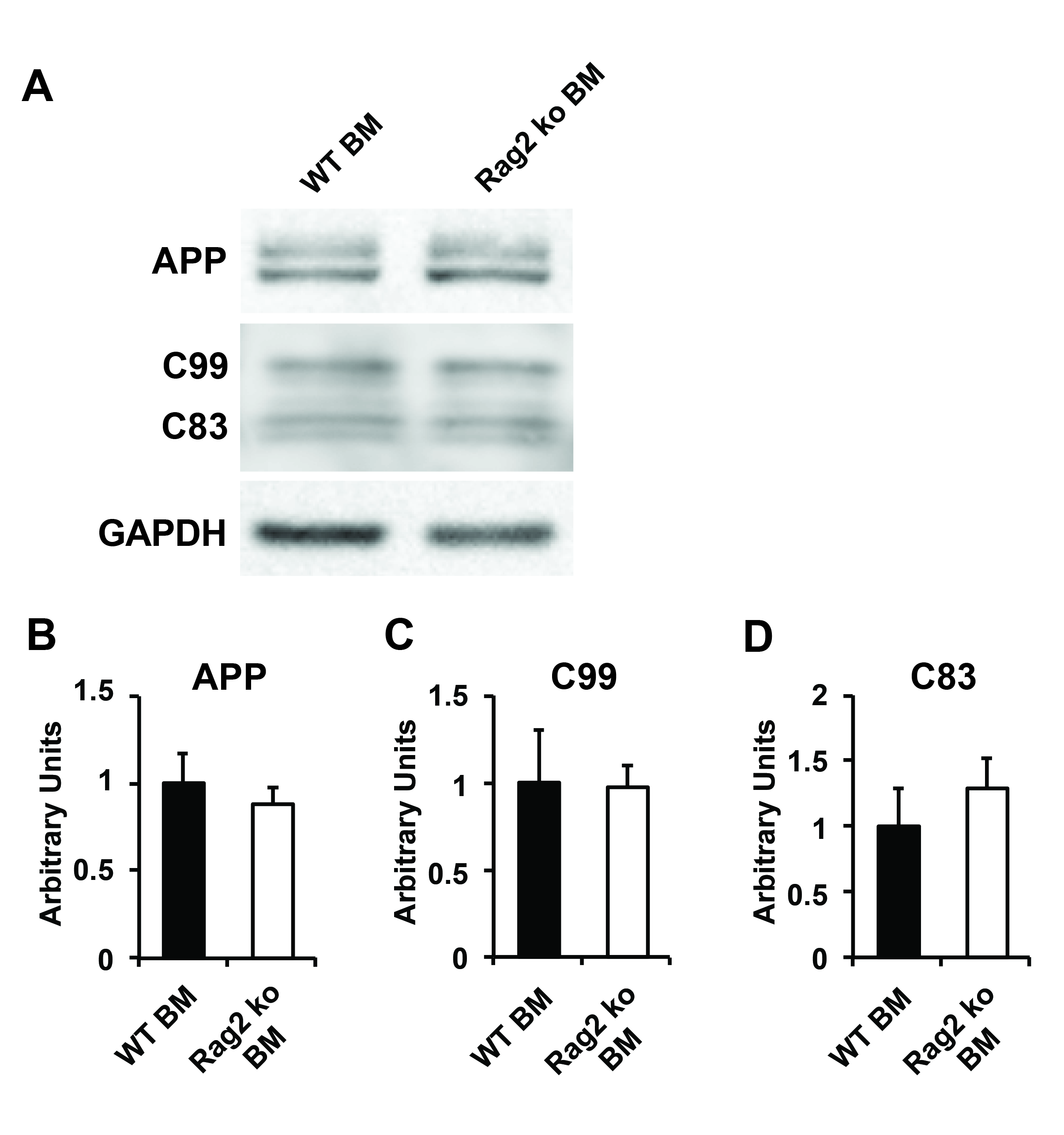

Supplement: Additional file 6: Figure S6. — No difference in APP processing. (A) Western blot analysis of cortical SDS extracts from 18-month-old PSAPP mice reconstituted with WT or Rag2 ko BM 6 months before sacrifice. Values were normalized to GAPDH. (B) Quantification reveals no significant differences in full length APP and APP C-terminal fragments (C99 and C83). n = 6 per group. (TIF 26355 kb) [file 40478_2015_251_MOESM6_ESM.tif]

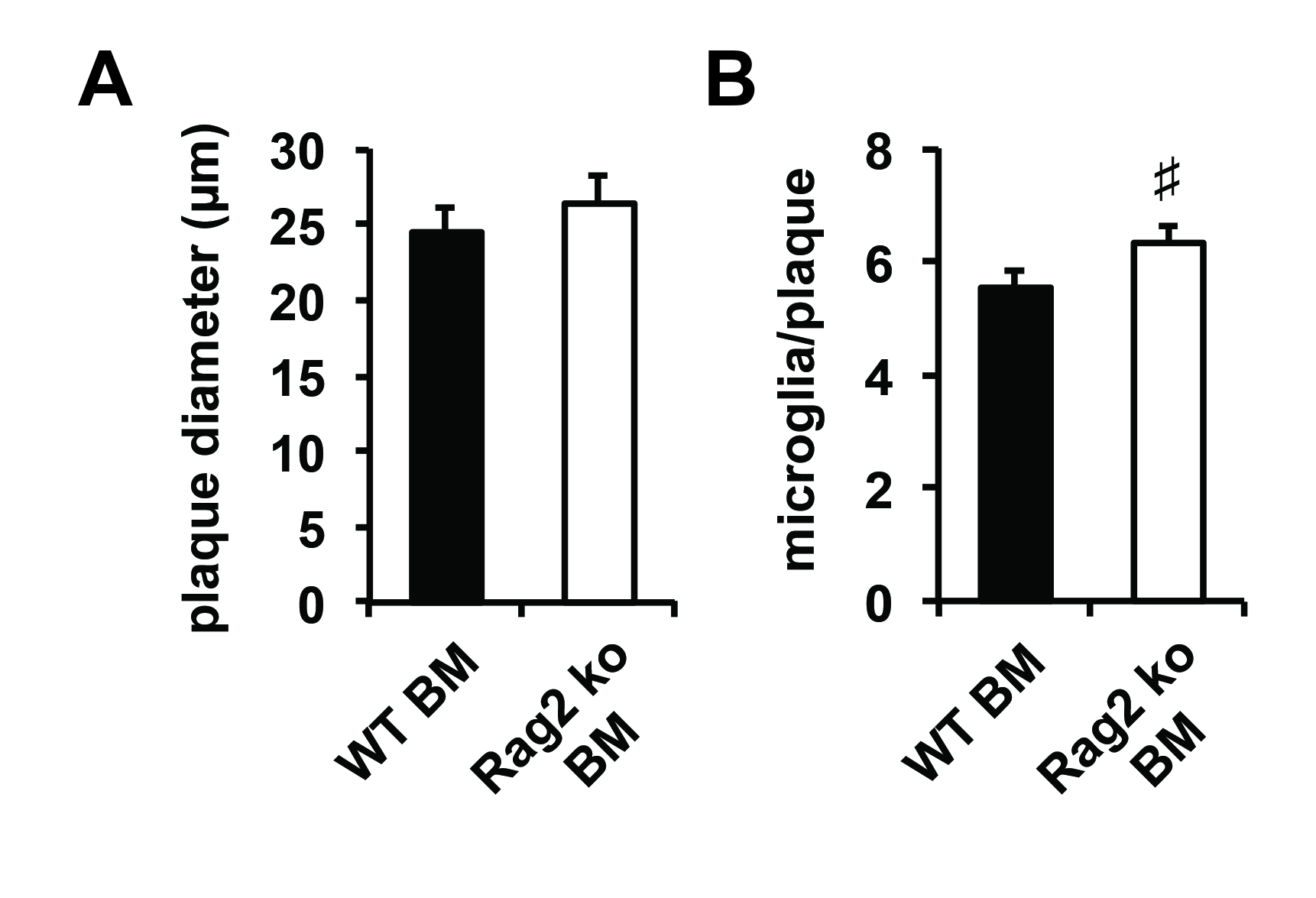

Supplement: Additional file 7: Figure S7. — Similar size of amyloid plaques analyzed by confocal microscopy. (A) Comparable average size of analyzed amyloid plaques between WT and Rag2 ko BM-reconstituted PSAPP mice at the age of 18 months. On average 12 Thioflavin S-stained amyloid plaques per mouse (n = 9 mice per group) were analyzed by laser scanning confocal microscopy. p = 0.711. (B) Trend towards an increased average number of Iba1-positive cells per plaque in the Rag2 ko BM-recipient PSAPP mice in comparison to the WT BM-reconstituted PSAPP mice at 18 months. Again, on average 12 Thioflavin S-stained amyloid plaques per mouse (n = 9 mice per group) were analyzed by laser scanning confocal microscopy. # p = 0.086. (TIF 8887 kb) [file 40478_2015_251_MOESM7_ESM.tif]
